# Prediction of the chance of successful immune tolerance induction in persons with severe hemophilia A and inhibitors: a clinical prediction model

**DOI:** 10.1016/j.rpth.2024.102580

**Published:** 2024-10-03

**Authors:** Ilja Oomen, Amal Abdi, Ricardo M. Camelo, Fábia M.R.A. Callado, Luany E.M. Carvalho, Ilenia L. Calcaterra, Manuel Carcao, Giancarlo Castaman, Jeroen C.J. Eikenboom, Kathelijn Fischer, Vivian K.B. Franco, Martijn W. Heymans, Frank W.G. Leebeek, David Lillicrap, Cláudia S. Lorenzato, Maria Elisa Mancuso, Davide Matino, Matteo N.D. Di Minno, Alex B. Mohseny, Johannes Oldenburg, Suely Meireles Rezende, Georges-Etienne Rivard, Natalia Rydz, Saskia E.M. Schols, Jan Voorberg, Karin Fijnvandraat, Samantha C. Gouw, International Genetic and clinical determinants for the outcome of immune tolerance induction study group

**Affiliations:** 1 Department of Pediatric Hematology, Amsterdam University Medical Centers location University of Amsterdam, Amsterdam, the Netherlands; 2 Department of Molecular Hematology, Sanquin Research, Amsterdam, the Netherlands; 3 Department of Internal Medicine, Faculty of Medicine, Universidade Federal de Minas Gerais, Belo Horizonte, Brazil; 4 Fundação de Hematologia e Hemoterapia de Pernambuco, Recife, Brazil; 5 Centro de Hematologia e Hemoterapia do Ceará, Fortaleza, Brazil; 6 Department of Clinical Medicine and Surgery, Federico II University, Naples, Italy; 7 Department of Pediatrics, Division of Hematology and Oncology, Hospital for Sick Children, Toronto, Ontario, Canada; 8 Department of Oncology, Center for Bleeding Disorders and Coagulation, Careggi University Hospital, Florence, Italy; 9 Department of Internal Medicine, Division of Thrombosis and Hemostasis, Leiden University Medical Center, Leiden, the Netherlands; 10 Department of Hematology, Center for Benign Hematology, Thrombosis and Hemostasis, Van Creveldkliniek, University Medical Center Utrecht, Utrecht, the Netherlands; 11 Centro de Hematologia e Hemoterapia de Santa Catarina, Florianópolis, Brazil; 12 Department of Epidemiology and Biostatistics, Amsterdam University Medical Centers, Vrije Universiteit University, Amsterdam, the Netherlands; 13 Department of Epidemiology and Data Science, Amsterdam Public Health Research Institute, Amsterdam University Medical Centers, University of Amsterdam, the Netherlands; 14 Department of Hematology, Erasmus University Medical Center, Rotterdam, the Netherlands; 15 Department of Pathology and Molecular Medicine, Queen’s University, Kingston, Ontario, Canada; 16 Coagulopathy Clinic, Hemocentro do Paraná, Curitiba, Brazil; 17 Department of Hematology, Center for Thrombosis and Hemorrhagic Diseases, Instituto di Ricovero e Cura a Carattere Scientifico Humanitas Research Hospital, Rozzano, Milan, Italy; 18 Department of Health Research Methods, Evidence, and Impact, McMaster University, Hamilton, Ontario, Canada; 19 Department of Pediatrics, Leiden University Medical Center, Leiden, the Netherlands; 20 Institute of Experimental Hematology and Transfusion Medicine, University Hospital Bonn, Medical Faculty, University of Bonn, Bonn, Germany; 21 Molecular Diagnostic Laboratory, Centre Hospitalier Universitaire Sainte-Justine, Montréal, Québec, Canada; 22 Department of Pediatrics, Division of Hematology-Oncology, Montréal University, Centre Hospitalier Universitaire Sainte-Justine, Montréal, Québec, Canada; 23 Department of Hematology and Hematologic Malignancies, Foothills Medical Center, Calgary, Alberta, Canada; 24 Department of Hematology, Radboud University Medical Center, Nijmegen, the Netherlands; 25 Hemophilia Treatment Center Nijmegen-Eindhoven-Maastricht, Nijmegen, the Netherlands

**Keywords:** factor VIII, hemophilia A, immune tolerance, probability, treatment outcome

## Abstract

**Background:**

Inhibitor eradication to restore factor (F)VIII efficacy is the treatment goal for persons with severe hemophilia A (HA) and inhibitors. Immune tolerance induction (ITI) is demanding and successful in about 70% of people. Until now, it has remained difficult to quantify the probability of ITI success or failure, complicating the decision to initiate or not initiate ITI. Estimating the individual chance of ITI success allows clinicians, patients, and their families to support shared decision-making.

**Objectives:**

We aimed to identify clinical predictors of ITI success and to develop a clinical prediction model to estimate the chance of successful ITI in persons with severe HA.

**Methods:**

This multicenter study included persons with severe HA who received ITI. Clinical data were collected. Successful ITI was defined by a negative inhibitor titer and an adequate response to FVIII concentrates. A multivariable logistic regression model was developed. Model performance and internal validation were performed.

**Results:**

Of 206 participants with a median age of 19.8 months (IQR, 12.1-38.8) at ITI start, 148 (71.8%) achieved ITI success. Our clinical prediction model included 4 predictors of ITI success: cumulative number of FVIII exposure days at inhibitor development, peak inhibitor titer, ethnicity, and *F8* mutation type. The C statistic was 0.801 (95% CI, 0.70-0.87).

**Conclusion:**

In our study, including 206 people with severe HA and inhibitors, we developed a clinical prediction model to estimate the chance of successful ITI. After future external validation, this clinical prediction model may be useful for informing clinicians and families.

## Essentials

• Predicting the chance of immune tolerance induction success may guide shared decision-making.
• Prediction models were developed to estimate the chance of immune tolerance induction success.
• Predictors were number of exposure days, peak inhibitor titer, ethnicity, and *F8* mutation type.
• After external validation, our prediction models may be useful in shared decision-making.

## 1 Introduction

Hemophilia A (HA) is a hereditary bleeding disorder caused by a deficiency in the coagulation factor (F)VIII. The hemophilia severity is determined by the residual plasma FVIII activity (FVIII:C) and classified as severe (FVIII plasma activity below 1 IU/dL), moderate (FVIII plasma activity between 1 and 5 IU/dL), and mild (FVIII plasma activity between 5 and 40 IU/dL). In case of bleeding, FVIII concentrates are administered intravenously to adequately treat bleeding. Persons with hemophilia can be treated prophylactically with intravenous FVIII infusions or nonreplacement therapy (emicizumab) to prevent bleeds. However, the most severe complication of FVIII replacement therapy is the development of FVIII-neutralizing antibodies, called inhibitors, which unfortunately occur in about 30% of persons with severe HA [1]. Inhibitors develop predominantly in young children (median age of 15 months) after a median of 12 to 15 FVIII exposure days [2].

In the current evolving treatment era of hemophilia, inhibitor eradication is still the treatment goal, allowing them to return to further treatment with FVIII concentrates. The only effective therapy to eradicate inhibitors is immune tolerance induction (ITI), which consists of frequent infusions of high doses of FVIII concentrates for several months to years [3,4]. ITI is a demanding, burdensome, and very expensive therapy. It is successful in about 70% of those treated. In contrast, in the remaining 30% of treated individuals, the inhibitor persists [5]. Therefore, there is a strong need for the identification of individuals with a high or low chance of successful ITI to adequately inform patients, parents, and clinicians of the best possible treatment option. Indeed, some families and their physicians opt to refrain from initiating ITI, especially if their risk of failing is considered to be high. This is reasonable, as alternative treatment options are available, such as the humanized bispecific antibody emicizumab [6]. However, up to now, it has not yet been possible to predict the individual chance/risk of ITI success or failure.

Previous reports have identified possible predictor variables of ITI success [[7], [8], [9]]. In a recent meta-analysis, the strongest predictors of ITI success were peak inhibitor titer below 100 Bethesda Units (BU)/mL (odds ratio [OR], 1.7; 95% CI, 1.4-2.1), inhibitor titer at the start of ITI below 10 BU/mL (OR, 1.8; 95% CI, 1.4-2.3), and peak inhibitor titer during ITI below 100 BU/mL (OR, 2.7; 95% CI, 1.9-3.8). These peak inhibitor titers usually increase in the first period after starting ITI and subsequently slowly decrease in case of successful ITI [10].

To date, it has remained difficult to quantify the probability of ITI success or failure, complicating the decision to initiate or not initiate ITI. In shared decision-making, it may be helpful to consult a clinical prediction model that could estimate the individual probability of successful ITI to tailor therapies. Therefore, we aimed to develop 2 clinical prediction models: (1) to estimate the chance of successful ITI before ITI starts, and (2) to reestimate the chance of ITI success after 6 months on ITI to consider ITI continuation after the initial inhibitor titer increase. This estimate will support the decision on whether or not to initiate ITI and whether or not to continue ITI after 6 months of ITI.

## 2 Methods

### 2.1 Study design and population

This observational, retrospective, multicenter cohort study included children and adults with severe HA (FVIII:C < 1 IU/dL) and a history of inhibitors who received ITI in one of the hemophilia treatment centers. Participants were recruited at hemophilia treatment centers in Brazil, Canada, the Netherlands, Germany, and Italy between 2015 and 2023. All participating centers are listed in Supplementary Table S1. Persons were excluded from the current analysis if hemophilia severity was unknown, if ITI was still ongoing, or if the cumulative number of exposure days at inhibitor development exceeded 150 days. No limit was defined for age at inhibitor development.

Ethical approval was obtained from the institutional boards of each hemophilia treatment center. The study is registered under number NL53406.018.15. Written informed consent was obtained from all participants or parents/guardians in the case of minors, according to the Declaration of Helsinki.

### 2.2 Defining outcome and predictor variables

As formal FVIII *in vivo* recovery and FVIII half-life assessment were only performed in a subset of participants, we defined ITI success as having a (1) negative inhibitor titer and (2) clinical response to standard doses of FVIII concentrates [11]. ITI failure was defined as failure to achieve success. Negative inhibitor titer was defined as the absence of an inhibitor titer, according to the local laboratory, in 2 consecutive assays. Clinical response to standard doses of FVIII concentrates was defined as a response that allowed bleeding to be prevented or treated. ITI regimen was defined as FVIII concentrate infusions at a dose ≥25 IU/kg at least thrice weekly.

Candidate predictors were selected based on previously identified predictors of ITI outcome from available literature and the availability of the predictor in the data set [7,12]. A total of 12 clinical variables were considered as potential predictors, including *F8* mutation type, ethnicity, family history of inhibitor development, age at inhibitor development, inhibitor titer at detection, cumulative number of FVIII exposure days at inhibitor development, inhibitor titer prior to ITI start, peak inhibitor titer, age at ITI start, time interval between inhibitor development and ITI start, FVIII product type, and dose at ITI start.

*F8* mutation type was categorized into large deletions or other mutations, as large deletion was previously associated with a high risk of inhibitor development and ITI failure [13,14]. Ethnicity was categorized into Hispanic/Latino or other descent, as previous studies suggested the highest risk of inhibitor development and ITI failure in persons with Hispanic/Latino ethnicity [15,16]. FVIII product was categorized into recombinant FVIII and plasma-derived FVIII. Family history of inhibitors was analyzed as a categorical variable as follows: (a) negative family history of HA, (b) positive family history of HA and positive family history of inhibitors, (c) positive family history of HA and negative family history of inhibitors, or (d) unknown family history of HA or inhibitors [17]. Other variables were analyzed as continuous variables.

### 2.3 Data collection

Clinical data were collected using study-specific case report forms. The following clinical data were collected: date of birth, baseline FVIII:C, von Willebrand factor activity level, von Willebrand factor antigen level, *F8* mutation type, ethnicity, comorbidities including autoimmune disease or HIV, family history of HA and inhibitors, age at inhibitor development, inhibitor titer at development, the cumulative number of FVIII exposure days at inhibitor development, inhibitor titer before ITI start, peak inhibitor titer ever measured, ITI regimen including product, dose, and frequency, concomitant use of immunomodulatory medication, presence of a major infection during ITI, date of first negative inhibitor titer after ITI start, date of normal FVIII recovery after ITI start, date of normal FVIII half-life after ITI start, date of clinical response to FVIII after negative inhibitor titer, and inhibitor relapse.

### 2.4 Data analysis

Baseline characteristics were summarized by numbers and proportions using descriptive statistics. Continuous variables were reported as medians with IQR using descriptive statistics. The numbers and proportions of the study outcome, eg, the cumulative incidence of success and failure, were calculated.

Pearson’s chi-square test was used to compare categorical variables between ITI outcome groups. In the case of small numbers per determinant group, Fisher’s exact test was used instead (Table 1). Independent samples median test was used to compare medians with IQRs between ITI outcome groups.

**Table 1:** Baseline characteristics.

Characteristic	Total cohort (*N* = 206) *n* (%*N*)	ITI outcome		
		Success (*n* = 148) *n* (%)	Failure (*n* = 58) *n* (%)	*P* value
**Patient characteristics**				
*F8* mutation type				.077
Intron 22 inversion	106 (51.5)	77 (72.6)	29 (27.4)	
Intron 1 inversion	6 (2.9)	6 (100.0)	0 (0.0)	
Other inversion	1 (0.5)	1 (100.0)	0 (0.0)	
Large deletion	13 (6.3)	4 (30.8)	9 (69.2)	
Nonsense mutation	29 (14.1)	21 (72.4)	8 (27.6)	
Small deletion or insertion	15 (7.3)	11 (73.3)	4 (26.7)	
Missense mutation	13 (6.3)	10 (76.9)	3 (23.1)	
Splice site mutation	2 (1.0)	2 (100.0)	0 (0.0)	
*Missing, no mutation found/not determined*	*21 (10.2)*	*16 (76.2)*	*5 (23.8)*	
Ethnicity				.20
Caucasian	157 (76.2)	115 (73.2)	42 (26.8)	
Hispanic/Latino	22 (10.7)	12 (54.5)	10 (45.5)	
African/African American/African-Caribbean	8 (3.9)	7 (87.5)	1 (12.5)	
Arab/Middle Eastern	7 (3.4)	5 (71.4)	2 (28.6)	
Asian	5 (2.4)	5 (100.0)	0 (0.0)	
Other^a^	4 (1.9)	3 (75.0)	1 (25.0)	
*Missing*	*3 (1.5)*	*1 (33.3)*	*2 (66.7)*	
von Willebrand factor activity (IU/mL), median (IQR)	100.0 (78.0-136.0)	100.0 (78.0-131.8)	103.0 (76.0-139.0)	.806
*Missing*	*107 (51.9)*	*68 (63.6)*	*39 (36.4)*	
von Willebrand factor antigen (IU/mL), median (IQR)	108.5 (86.5-142.0)	114.0 (90.3-142.5)	100.5 (77.5-141.3)	.47
*Missing*	*98 (47.6)*	*62 (63.3)*	*36 (36.7)*	
HIV status				.446^b^
HIV infected	8 (3.9)	7 (87.5)	1 (12.5)	
*Missing*	*7 (3.4)*	*5 (71.4)*	*2 (28.6)*	
Family history				.306^b^
HA, inhibitors	26 (12.6)	17 (65.4)	9 (34.6)	
HA, no inhibitors	56 (27.2)	42 (75.0)	14 (25.0)	
No HA	107 (51.9)	75 (70.1)	32 (29.9)	
*Unknown family history of HA*	*17 (8.3)*	*14 (82.4)*	*3 (17.6)*	
**Characteristics at inhibitor development**				
Age at inhibitor development (mo), median (IQR)	19.8 (12.1-38.8)	20.0 (12.2-41.9)	19.6 (11.3-32.5)	.87
*Missing*	*18 (8.7)*	*12 (66.7)*	*6 (33.3)*	
Cumulative number of FVIII exposure days at inhibitor development, median (IQR)	12.0 (8.0-22.0)	14.0 (9.0-25.0)	9.0 (5.0-12.5)	.01
*Missing*	*83 (40.3)*	*53 (63.9)*	*30 (31.1)*	
Inhibitor titer at detection (BU/mL), median (IQR)	5.1 (1.4-20.0)	4.2 (1.5-16.0)	14.5 (1.4-46.8)	.06
*Missing*	*17 (8.3)*	*9 (52.9)*	*8 (47.1)*	
**Peak inhibitor titers**				
Peak inhibitor titer ever measured (BU/mL), median (IQR)	51.3 (9.8-200.0)	18.4 (5.7-96.5)	188.5 (96.7-726.3)	<.001
*Missing*	*10 (4.9)*	*4 (40.0)*	*6 (60.0)*	
Peak inhibitor titer ever measured before ITI start (BU/mL), median (IQR)	20.4 (7.9-76.6)	13.1 (6.1-46.4)	92.9 (48.0-470.0)	<.001
*Missing*	*94 (45.6)*	*60 (63.8)*	*34 (36.2)*	
Peak inhibitor titer ever measured after ITI start (BU/mL), median (IQR)	82.0 (15.0-327.6)	36 (4.2-188.6)	179.0 (82.0-486.5)	<.001
*Missing*	*78 (37.9)*	*69 (88.5)*	*9 (11.5)*	
Peak inhibitor titer ever measured until 6 mo on ITI (BU/mL), median (IQR)	46.5 (10.2-193.5)	18.2 (6.1-95.0)	169.6 (74.5-736.0)	<.001
*Missing or people <6 mo on ITI treatment*	*45 (21.8)*	*19 (42.2)*	*26 (57.8)*	
**Pre-ITI characteristics**				
Last inhibitor titer measured before ITI start (pre-ITI titer; BU/mL), median (IQR)	5.6 (2.2-10.1)	4.5 (2.0-11.3)	13.7 (4.1-38.8)	.001
*Missing*	*17 (8.3)*	*9 (52.9)*	*8 (47.1)*	
Interval between inhibitor development and ITI start (wk), median (IQR)	16.0 (2.0-59.8)	12.5 (1.3-54.8)	23.5 (6.0-89.0)	.22
*Missing*	*14 (6.8)*	*8 (57.1)*	*6 (42.9)*	
**Characteristics at ITI start**				
Age at ITI start (mo), median (IQR)	26.1 (15.1-63.0)	26.6 (14.9-66.2)	26.0 (15.7-59.2)	0.85
*Missing*	*11 (5.3)*	*8 (72.7)*	*3 (27.3)*	
FVIII product at ITI start				.26^b^
Recombinant FVIII	123 (59.7)	85 (69.1)	38 (30.9)	
Plasma-derived FVIII	78 (37.9)	60 (76.9)	18 (23.1)	
*Missing*	*5 (2.4)*	*3 (60.0)*	*2 (40.0)*	
ITI dose at ITI start in IU/kg/d, median (IQR)	42.9 (21.4-200.0)	54.4 (21.4-200.0)	36.0 (21.4-200.0)	.785
*Missing*	*7 (3.4)*	*4 (57.1)*	*3 (42.9)*	

BU, Bethesda Units; FVIII, factor VIII; HA, hemophilia A; ITI, immune tolerance induction; IU, international units.

a Native-American (*n* = 2), Hispanic and Caucasian (*n* = 1), Mexican, Hispanic, and Caucasian (*n* = 1).

b Fisher’s exact test was performed due to the small patient numbers.

Natural spline and restricted cubic spline models were used to assess the linearity of the associations of continuous variables with ITI outcome [18,19].

In order to quantify the probability of ITI success or failure, we created 2 nomograms that allow us to estimate the chance of successful ITI in individuals with severe HA and inhibitors. Two different clinical prediction models were fitted using logistic regression at 2 time points according to the transparent reporting of multivariable prediction models for individual prognosis or diagnosis statement [20]. Model A was developed to be used at ITI initiation. A second prediction model (model B) was developed to reestimate the chance of ITI success after 6 months on ITI because inhibitor titers may increase after starting ITI. Associations between each potential predictor variable of ITI success were assessed using univariate logistic regression analysis (Supplementary Table S2). Predictor variables were included in the final multivariable logistic regression models if the *P* value in multivariable analysis remained below .157 [21]. Predictor variables included in the final multivariable logistic regression models are reported in Table 2.

**Table 2:** Specific characteristics of immune tolerance induction and immune tolerance induction outcome.

Characteristic	Total cohort (*N* = 206) *n* (%*N*)	ITI outcome	
		Success (*n* = 148) *n* (%)	Failure (*n* = 58) *n* (%)
ITI duration (mo), median (IQR)	32.5 (19.3-52.0)	27.0 (15.3-42.0)	43.5 (33.3-75.3)
*Missing*	*38 (18.4)*	*28 (73.7)*	*10 (26.3)*
Concomitant use of immunomodulatory drugs during ITI			
Yes	11	7 (46.7)	8 (53.3)
*Missing*	*11 (5.3)*	*7 (63.6)*	*4 (36.4)*
Presence of a port infection during ITI			
Yes	18	9 (50.0)	9 (50.0)
*Missing*	*26 (12.6)*	*16 (61.5)*	*10 (38.5)*
Negative inhibitor titer			
Yes	155	148 (95.5)	7 (4.5)
No	50	0 (0.0)	50 (100.0)
*Missing*	*1 (0.5)*	*0 (0.0)*	*1 (100.0)*
Normal FVIII recovery			
Yes	69	69 (100.0)	0 (0.0)
No	52	10 (19.2)	42 (80.8)
*Missing*	*85 (41.3)*	*69 (81.2)*	*16 (18.8)*
Normal FVIII half-life			
Yes	58	58 (100.0)	0 (0.0)
No	49	7 (14.3)	42 (85.7)
*Missing*	*99 (48.1)*	*83 (83.8)*	*16 (16.2)*
Relapse after successful ITI			
Yes	12	12 (100.0)	0 (0.0)
*Missing*	*29 (14.1)*	*29 (100.0)*	*0 (0.0)*

FVIII, factor VIII; ITI, immune tolerance induction.

### 2.5 Handling missing values

Missing values for any of the determinants were imputed with multiple imputations using predictive mean matching with 50 imputations with 50 iterations [22,23]. The total number of missing values per predictor is presented in Table 1. Trace plots were used to visually assess convergence (Supplementary Figure S2). Strip plots were created to compare the distribution of both imputed and nonimputed individual values (Supplementary Figure S3). All results were pooled using Rubin’s Rules (D1 method for categorical variables) [[24], [25], [26], [27], [28]]. Distribution before and after imputation of variables with more than 10% missing values are presented in Supplementary Table S3.

To evaluate the effect of imputation on ITI outcome for determinants with more than 10% missing data, we compared the univariable effects between these predictors and ITI outcome before imputation and after 5, 10, or 50 imputations (data not shown). As the results were comparable between the different numbers of imputations, we used 50 imputations with 50 iterations for further analysis, as this may achieve better estimates of SEs [23].

### 2.6 Evaluating model performance

The performance of the clinical prediction models was assessed with discrimination and calibration [17,19,29]. Discrimination is the level to which a model can distinguish between individuals achieving or not achieving the outcome. The discriminative power of each model was calculated with the C statistic, which can range from 0.5 (no discrimination) to 1 (perfect discrimination). Calibration refers to the degree to which predicted and observed outcomes are similar. The calibration of each model was reported visually in a calibration plot. Sensitivity, specificity, and positive and negative predictive values were calculated for different cutoff values of our models.

### 2.7 Internal validation using bootstrap resampling

To correct for overfitting of the model, a uniform shrinkage factor was estimated using the bootstrapping resampling method in which 250 bootstrap resampling runs were performed [17,[29], [30], [31]]. Subsequently, model coefficients were multiplied by the shrinkage factor, and the model intercept was reestimated with the shrunken coefficients.

### 2.8 Sensitivity analyses

A sensitivity analysis was performed in a subgroup of participants with age at inhibitor development below 50 months. As individuals who develop inhibitors as older children or adults represent a different patient population than the targeted people who develop inhibitors within the first 50 to 75 exposure days, we excluded the 20% of persons with the highest age at inhibitor development (Supplementary Table S4).

Another sensitivity analysis was performed to identify predictors for complete ITI success, according to the ITI outcome definitions defined by the international ITI study [9]. Complete ITI success was defined by (1) a negative inhibitor titer, (2) a normal FVIII recovery ≥ 66% of expected, (3) a normal FVIII half-life ≥ 6 hours after a 72-hour washout period, and (4) absence of anamnesis upon further FVIII exposure. Partial ITI success was defined as a negative inhibitor titer, but the presence of at least 1 of the following criteria: an FVIII recovery of <66% of expected, and/or FVIII half-life of <6 hours after a 72-hour washout period with clinical response to FVIII therapy without an anamnestic response. ITI failure was defined as failure to achieve complete or partial success. This sensitivity analysis included a subset of participants in whom all data required for the complete ITI outcome definition were available, including FVIII recovery and half-life (Supplementary Table S5).

### 2.9 Statistical packages

The data were prepared for analysis using SPSS statistics (IBM) version 28. Baseline characteristics were analyzed using SPSS statistics (IBM) version 28. Data and statistical analyses were performed using RStudio (Posit) version 4.2.1 using the packages mice and psfmi [32].

## 3 Results

A total of 224 persons were included. After exclusion of 18 subjects, the current study included 206 people with severe HA. Reasons for exclusions included ongoing ITI (*n* = 6), cumulative number of FVIII exposure days of more than 150 days (*n* = 4), treatment regimen not fulfilling ITI definition (*n* = 3), duplicate patient inclusion (*n* = 2), and unknown hemophilia severity (*n* = 2) or mild HA (*n* = 1).

Baseline characteristics are shown in Table 1. The majority (51.5%) of participants had an intron 22 inversion. Caucasian ethnicity was the most prevalent (76.2%). The median age at inhibitor development was 19.8 months (IQR, 12.1-38.8), and the median cumulative number of FVIII exposure days at inhibitor development was 12.0 days (IQR, 8.0-22.0). The median inhibitor titer at detection was 5.1 BU/mL (IQR, 1.4-20.0). A total of 148 (71.8%) persons achieved ITI success.

Differences in the distribution of baseline characteristics between individuals with ITI success and failure were compared (Table 1). Higher inhibitor titer prior to ITI start (pre-ITI titer) and peak inhibitor titers were observed in persons with ITI failure compared with ITI success (median, 13.7 vs 4.5 BU/mL; *P* = .001, and median, 188.5 vs 18.4 BU/mL; *P* < .001). In addition, a lower cumulative number of FVIII exposure days at inhibitor development was seen in persons with ITI failure than in those with ITI success (median, 9.0 vs 14.0 days; *P* = .01).

Specific characteristics for ITI and ITI outcomes are presented in Table 2.

### 3.1 Predictor variables of ITI success

Univariate associations between predictor variables and ITI success are shown in Supplementary Table S2. Predictor variables that remained significant predictors of successful ITI in multivariable analysis (*P* < .157) were included in the final multivariable logistic regression models.

The clinical prediction model for ITI success at ITI start (model A) included *F8* mutation type, ethnicity, cumulative number of FVIII exposure days at inhibitor development, and peak titer before ITI start (Table 3).

**Table 3:** Final logistic regression model.

Variable	Original coefficients	Bootstrap-adjusted coefficients^a^	SE	OR	95% CI	*P* value
**Model A – probability of ITI success at ITI start**						
*F8* mutation type						
Other mutation	*Ref*	*Ref*	*Ref*	*Ref*	*Ref*	*Ref*
Large deletion	−1.504	−1.259	0.707	0.22	0.05-0.90	.03
Ethnicity						
Other ethnicity	*Ref*	*Ref*	*Ref*	*Ref*	*Ref*	*Ref*
Hispanic/Latino	−0.960	−0.804	0.557	0.38	0.13-1.15	.08
CED at inhibitor development	0.043	0.035	0.026	1.04	0.99-1.10	.09
Peak inhibitor titer before ITI starts (BU/mL)	−0.002	−0.002	0.001	1.00	1.00-1.00	.039
**Model B – probability of ITI success at 6 mo on ITI**						
*F8* mutation type						
Other mutation	*Ref*	*Ref*	*Ref*	*Ref*	*Ref*	*Ref*
Large deletion	−1.469	−1.095	0.665	0.23	0.06-0.86	.027
Ethnicity						
Other ethnicity	*Ref*	*Ref*	*Ref*	*Ref*	*Ref*	*Ref*
Hispanic/Latino	−0.763	−0.569	0.535	0.47	0.16-1.34	.15
CED at inhibitor development	0.043	0.032	0.027	1.04	0.99-1.10	.12
Peak inhibitor titer before ITI starts until 6 mo on ITI (BU/mL)	−0.001	−0.000	0.000	1.00	1.00-1.00	.08

BU, Bethesda Units; CED, cumulative number of factor VIII exposure days; ITI, immune tolerance induction; OR, odds ratio; Ref, reference category.

a The shrinkage factor was used to obtain the bootstrap-adjusted coefficients (original coefficient ∗ shrinkage factor = bootstrap-adjusted regression coefficient). The shrinkage factor was 0.837 for model A and 0.746 for model B.

The clinical prediction model to be used after 6 months on ITI (model B) identified similar predictor variables of ITI success. However, in this model, the peak inhibitor titer included the peak inhibitor titer ever measured until 6 months on ITI (Table 3).

### 3.2 Model performance and internal validation

**Figure 1:**
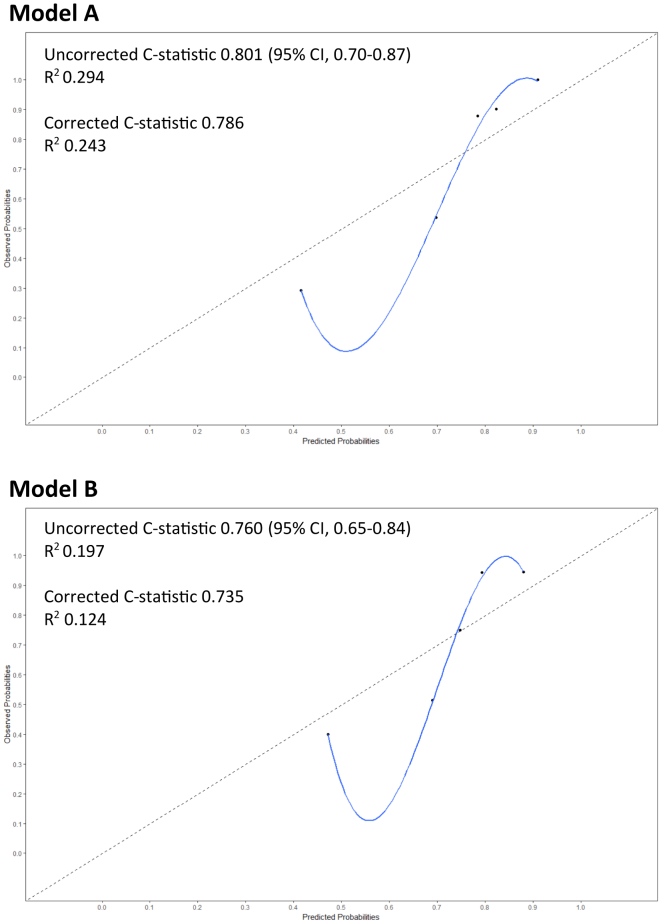
Calibration plots. These calibration plots can be interpreted as predicted (x-axis) and observed (y-axis) probability of immune tolerance induction success. Uncorrected C statistic represents the calibration of the prediction model when applied to the data set. The corrected C statistic shows the calibration adjusted for overfitting using bootstrap resampling (250 runs). If the predicted probability were equal to the observed probability, the line would have followed the diagonal dotted line.

Both models, A and B, were assessed for performance and internal validation. The calibration plots are presented in Figure 1. The shrunken regression coefficients are presented in Table 3. The discriminative ability of the multivariable analysis of models A and B was assessed using C statistics, which was 0.80 (95% CI, 0.70-0.87) and 0.76 (95% CI, 0.65-0.84), respectively (Figure 1). After bootstrap resampling, the corrected C statistics were 0.79 and 0.74, respectively (Figure 1). Table 4 shows the sensitivity, specificity, and positive and negative predictive values of models A and B for different model cutoff values. The negative predictive value was low when using the medium and high cutoff values and higher for the low cutoff value. Conversely, the positive predictive value was high for all 3 model cutoff values.

**Table 4:** Sensitivity, specificity, and positive and negative predictive values of the models for different model cutoff values.

Categories of predicted risk according to model	Sensitivity	Specificity	Positive predictive value	Negative predictive value
**Model A – probability of ITI success at ITI start**				
Low cutoff (10%)	0.90	0.50	0.82	0.66
Medium cutoff (25%)	0.26	0.97	0.95	0.34
High cutoff (40%)	0.12	1.00	1.00	0.31
**Model B – probability of ITI success at 6 mo on ITI**				
Low cutoff (10%)	0.97	0.21	0.75	0.73
Medium cutoff (25%)	0.48	0.85	0.88	0.40
High cutoff (40%)	0.11	0.96	0.88	0.31

The purpose of our study is to create a prediction model that can estimate the individual probability of ITI success or failure, which can be used in shared decision-making. It is important to mention that it is not the purpose of the current study to define a cutoff to start or refrain from ITI therapy.

ITI, immune tolerance induction.

### 3.3 Clinical application for using these models

**Figure 2:**
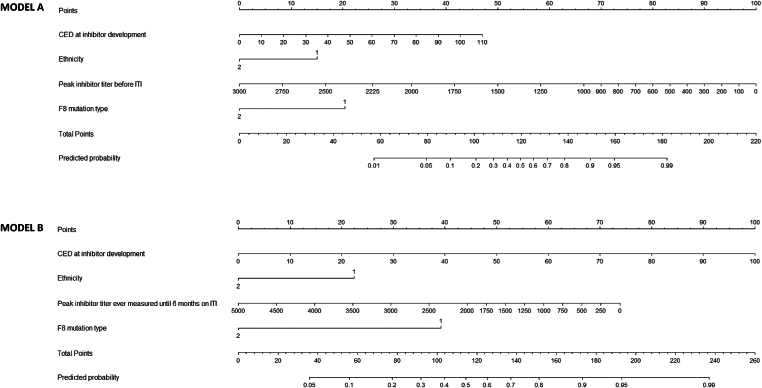
Nomograms. An illustration of the use of these nomograms. Ethnicity values: 1 = other ethnicities, 2 = Hispanic/Latino ethnicity. *F8* mutation type: 1 = other mutation, 2 = large deletion. Model A. A person with a large deletion in the *F8* gene (20 points, “points” scale on the upper bar), peak inhibitor titer before immune tolerance induction (ITI) start of 12.4 Bethesda Units (BU)/mL (95 points), Caucasian ethnicity (15 points), and 20 cumulative number of factor (F)VIII exposure days at inhibitor development (8 points) has a total score of (20 + 95 + 15 + 8 =) 138 points (second to lowest bar), corresponding to a predicted probability of 0.78 (lowest bar). Translating this to the clinic, this person has an estimated 78% chance of ITI success before ITI initiation. Model B. After initiating ITI, the peak inhibitor titer increased to 250 BU/mL. We reestimate the chance of ITI success at 6 months on ITI; a large deletion in the *F8* gene (39 points, “points” scale on the upper bar), peak inhibitor titer ever measured until 6 months on ITI of 200.0 BU/mL (72 points), Caucasian ethnicity (22 points), and 20 cumulative number of FVIII exposure days at inhibitor development (20 points). The total score is (39 + 72 + 22 + 20 =) 153 points (second to lowest bar), corresponding to a predicted probability of 0.80 (lowest bar). Translating this to the clinic, this person has an estimated 80% chance of ITI success at 6 months on ITI. CED, cumulative number of FVIII exposure days.

In order to estimate the individual chance of ITI success at ITI initiation and after 6 months of ITI, nomograms were created using regression coefficients of the prediction models (Figure 2). An illustration of the use of these nomograms is presented in the subheading of the figure.

### 3.4 Sensitivity analyses

Baseline characteristics of the subgroup of participants with inhibitor development below 50 months of age and the subgroup of participants with available data on complete success are presented in Supplementary Tables S4 and S5. Characteristics were comparable with those of the total cohort (Table 1).

In the sensitivity analysis among participants with inhibitor development below 50 months of age, the prediction model of ITI success at ITI initiation showed similar predictor variables as in the total population (corrected C statistic 0.79, Supplementary Figure S1A and Supplementary Table S6A). At 6 months of ITI, the prediction model of ITI success in this subgroup included only *F8* mutation, the cumulative number of FVIII exposure days at inhibitor development, and peak inhibitor titer before ITI start until 6 months on ITI as predictor variables and did not include ethnicity in the final model (corrected C statistic 0.75, Supplementary Figure S1B and Supplementary Table S6B).

In the sensitivity analyses on the subgroup of patients with complete data to assess complete success, the prediction model for ITI success at ITI initiation included age at inhibitor development, ethnicity, and peak inhibitor titer before ITI start as predictor variables of ITI success at ITI start (corrected C statistic 0.65, Supplementary Figure S1C and Supplementary Table S6C).

## 4 Discussion

This international multicenter study included 206 persons with severe HA who underwent ITI in the past for inhibitor eradication. Using multivariable logistic regression analysis, we developed clinical prediction models to assess the probability of ITI success at ITI initiation and after 6 months on ITI. These prediction models identified 4 predictor variables of ITI success, including the number of cumulative FVIII exposure days at inhibitor development, peak inhibitor titer, ethnicity, and *F8* mutation type. The model performance was good. It is important to mention that the purpose of our study is to create clinical prediction models that can estimate the individual probability of ITI success or failure, which can be used in shared decision-making, and not to define a cutoff to start or refrain from ITI therapy.

Although the underlying immunologic mechanism of ITI is not yet fully understood, ITI is proposed to rely on T cell exhaustion through overstimulation and subsequent T cell anergy or apoptosis, inhibition of FVIII-specific memory B cells, and generation of anti-idiotypic antibodies [[33], [34], [35]]. In that regard, it is understandable that it may be more difficult to dampen a highly activated immune system in individuals with high-titer inhibitors, resulting in a higher risk of ITI failure. Indeed, the recent meta-analysis of our research group reported the highest probabilities of ITI success in persons with peak inhibitor titers during ITI below 100 BU/mL (pooled OR, 2.66; 95% CI, 1.9-3.8) [7]. This is in line with our current results.

Large deletions have previously been reported to constitute the *F8* mutation type with the highest risk for developing inhibitors in persons with severe HA (pooled OR, 14.8; 95% CI, 7.8-28.1) [13,36]. Indeed, mutations that are expected to cause a complete absence of FVIII are associated with the highest risk of inhibitor development. Persons with an intron 22 inversion, for example, express the entire FVIII protein as 2 polypeptide chains [37]. It has been proposed that intracellular production of portions of the endogenous FVIII protein may lead to some degree of tolerance to exogenous FVIII proteins and reduce the risk of inhibitor development [13]. Perhaps this reduced activation of the immune system could also explain the higher chance of ITI success in persons with other *F8* mutation types than large deletions. Other genes, especially immune response genes, may also play an important role in ITI outcome. To address this, we have additionally performed a large genetic study investigating more than 760,000 single nucleotide polymorphisms in different immune response genes. We only identified large deletions in the *F8* gene as a predictor for ITI failure (unpublished data). Nevertheless, we hope that we can pool different cohorts in the future to increase the power of the study, which will hopefully allow us to identify additional genes involved in this immune response.

In Brazil, skin color is reported instead of ethnicity. We classified brown as Hispanic/Latino, black as African/Caribbean, yellow as Asian, and white as Caucasian ethnicity. However, Caucasian classification likely does not reflect Brazilian Whites. In addition, brown skin color may represent a mixed ethnicity [38]. Nineteen of 22 (86.4%) participants with Hispanic/Latino ethnicity were from Brazil, of whom 10 failed ITI. Previous studies reported an increased risk of inhibitor development among persons of African/Caribbean or Hispanic descent [15,16,39]. Therefore, it is hypothesized that race and ethnicity may also impact ITI outcome. Indeed, in both our multivariable clinical prediction models, Hispanic/Latino ethnicity was associated with a higher risk of ITI failure. *F8* haplotypes have been suggested to explain this increased risk of inhibitor development between different ethnical groups. However, conflicting data are reported in the literature [40,41]. We considered differences in care and care access; however, for all participants, we used similar definitions for ITI regimen and ITI outcome. Importantly, only a small number of participants with a Hispanic/Latino ethnicity were included. Therefore, these results should be interpreted with caution, and larger studies are needed to confirm this association.

Other clinical variables were not clearly associated with ITI outcome. The time interval between inhibitor detection and ITI start was not associated with ITI outcome, which is in line with data published by Camelo et al. [11]. In addition, ITI outcome was not predicted by FVIII dose and frequency at ITI start, which is in line with the randomized dose comparison international ITI study [9]. Furthermore, although the Survey of Inhibitors in Plasma-Product Exposed Toddlers (SIPPET) trial reported an increased risk of inhibitor development in persons treated with recombinant FVIII, compared with plasma-derived FVIII, we found no association between FVIII product at ITI start and ITI outcome [42].

Strengths of the current study include constructive multiple imputation methods that provide valid results (Supplementary Figures S2 and S3, and Supplementary Table S3) [22,23] and sensitivity analyses to assess the robustness of our findings. Individuals who develop an inhibitor at an older age may reflect a different population than the targeted population of persons who develop inhibitors within the first 50 to 75 exposure days. Our sensitivity analysis in the subgroup of participants with age at inhibitor development below 50 months showed similar predictor variables for ITI success. We further performed a sensitivity analysis using a stricter definition of ITI success, as described in the international ITI study by Hay et al. [9].

A limitation of our study includes missing data on FVIII recovery and FVIII half-life in about 40% of our cohort. The sensitivity analysis for complete ITI success identified 3 predictors, including age at inhibitor development, ethnicity, and peak inhibitor titer before ITI start, and did not include a cumulative number of FVIII exposure days before inhibitor development and *F8* mutation type. The small sample size of this subcohort may explain the different results. Alternatively, the clustering of partial responders into the failure group may explain the difference in predictor variables included in this analysis compared with other analyses. Second, 2 predictor variables had more than 10% missing data. Although data imputation and pooling approaches were performed in a valid manner, we cannot fully exclude that the observed effects of these variables on ITI outcomes may have been influenced by the imputed data. Third, 4 predictor variables, including a cumulative number of FVIII exposure days at inhibitor development, pre-ITI inhibitor titer, inhibitor titer at detection, and peak inhibitor titers, were not corrected for nonlinearity in the multivariable logistic regression models as this caused a substantial reduction of model validation, probably due to introducing overfitting by applying flexible cubic spline models in these data. Fourth, due to the rapidly changing landscape in hemophilia care, ITI has changed since the first patient inclusions in this study, with the currently concomitant use of emicizumab. Both ITI interruptions and additional FVIII treatment due to intercurrent bleedings (in time of tissue damage) are suggested as negative predictors of ITI outcome; however, these are less present in the current hemophilia treatment landscape [43,44]. Thus, emicizumab may have a positive effect on ITI success rates; however, we assume that the identified predictors will not be different in current prospective studies. Lastly, we had only clinical data and DNA available; therefore, we were unable to also include immunologic markers, such as suggested markers for ITI outcome, like anti-FVIII immunoglobulin G levels or programmed cell death protein 1-exhaustion ratio, during the course of ITI [35,45].

At this moment, there is an ongoing debate among clinicians about whether ITI should be initiated in people with HA and inhibitors since emicizumab is available. We highlight the importance of inhibitor eradication to avoid the complications associated with a lifelong inhibitor due to ineffective treatment of bleeding. Therefore, it can be argued that ITI should be attempted in all persons with HA and inhibitors unless the estimated chance of ITI success is very low. Until now, it has remained difficult to quantify the probability of ITI success or failure. We have developed clinical prediction models that estimate the chance of successful ITI in individuals with severe HA and inhibitors. After future external validation, the estimation of the individual chance of ITI success allows it to support clinicians, patients, and their families in shared decision-making.
