# Supplementary material for: Prediction of the chance of successful immune tolerance induction in persons with severe hemophilia A and inhibitors: a clinical prediction model
Source: Res Pract Thromb Haemost. 2024 Oct 3;8(7):102580. doi: 10.1016/j.rpth.2024.102580 (PMC11570954; doi:10.1016/j.rpth.2024.102580)
Supplement: Supplementary material [file mmc1.docx]

**Supplementary material of “Prediction of the chance of successful immune tolerance induction in persons with severe hemophilia A and inhibitors: a clinical prediction model”.**

Ilja Oomen^1,2^, Amal Abdi^1^, Ricardo M. Camelo^3^, Fábia M.R.A. Callado^4^, Luany E.M. Carvalho^5^, Ilenia L. Calcaterra^6^, Manuel Carcao^7^, Giancarlo Castaman^8^, Jeroen C.J. Eikenboom^9^, Kathelijn Fischer^10^, Vivian K.B. Franco^11^, Martijn W. Heymans^12,13^, Frank W.G. Leebeek^14^, David Lillicrap^15^, Cláudia S. Lorenzato^16^, Maria Elisa Mancuso^17^, Davide Matino^18^, Dario M.N. Di Minno^6^, Alex B. Mohseny^19^, Johannes Oldenburg^20^, Suely Meireles Rezende^3^, Georges-Etienne Rivard^21,22^, Natalia Rydz^23^, Saskia E.M. Schols^24,25^, Jan Voorberg^2,26^, Karin Fijnvandraat^1,2^, Samantha C Gouw^1,27^, on behalf of the International GO-ITI study group.

**Affiliations**

1. Amsterdam UMC location University of Amsterdam, Department of Pediatric Hematology, Meibergdreef 9, Amsterdam, the Netherlands
2. Department of Molecular Hematology, Sanquin Research, Amsterdam, the Netherlands
3. Department of Internal Medicine, Faculty of Medicine, Universidade Federal de Minas Gerais, Belo Horizonte, Brazil
4. Fundação de Hematologia e Hemoterapia de Pernambuco (HEMOPE), Recife, Brazil
5. Centro de Hematologia e Hemoterapia do Ceará (HEMOCE), Fortaleza, Brazil
6. Department of Clinical Medicine and Surgery, Federico II University, Naples, Italy
7. Department of Pediatrics, Division of Hematology and Oncology, Hospital for Sick Children, Toronto, Canada
8. Department of Oncology, Center for Bleeding Disorders and Coagulation, Careggi University Hospital, Florence, Italy
9. Department of Internal Medicine, Division of Thrombosis and Hemostasis, Leiden University Medical Center, Leiden, the Netherlands
10. Department of Hematology, Center for Benign Hematology, Thrombosis and Hemostasis, Van Creveldkliniek, University Medical Center Utrecht, Utrecht, the Netherlands
11. Centro de Hematologia e Hemoterapia de Santa Catarina (HEMOSC), Florianópolis, Brazil
12. Department of Epidemiology and Biostatistics, Amsterdam Public Health Research Institute, Amsterdam University Medical Centers, VU University, Amsterdam, the Netherlands
13. Department of Epidemiology and Data Science, Amsterdam University Medical Centers, University of Amsterdam, the Netherlands
14. Department of Hematology, Erasmus University Medical Center, Rotterdam, the Netherlands
15. Department of Pathology and Molecular Medicine, Queen’s University, Kingston, Ontario, Canada
16. Coagulopathy Clinic, Hemocentro do Paraná (HEMEPAR), Curitiba, Brazil
17. Department of Hematology, Center for Thrombosis and Hemorrhagic Diseases, IRCCS Humanitas Research Hospital, Rozzano, Milan, Italy
18. Department of Health Research Methods, Evidence, and Impact (HEI), McMaster University, Hamilton, Ontario, Canada
19. Department of Pediatrics, Leiden University Medical Center, Leiden, the Netherlands
20. Institute of Experimental hematology and Transfusion Medicine, University Hospital Bonn, Medical Faculty, University of Bonn, Bonn, Germany
21. Molecular Diagnostic Laboratory, CHU Sainte-Justine, Montréal, Québec, Canada
22. Department of Pediatrics, Division of Hematology-Oncology, Montréal University, CHU Sainte-Justine, Montréal, Québec, Canada
23. Department of Hematology and Hematologic Malignancies, Foothills Medical Center, Calgary, Canada
24. Department of Hematology, Radboud university medical center, Nijmegen, the Netherlands
25. Hemophilia Treatment Center Nijmegen-Eindhoven-Maastricht, Nijmegen, the Netherlands
26. Department of Vascular Medicine, Amsterdam Cardiovascular Sciences, Amsterdam University Medical Centers, University of Amsterdam, Amsterdam, the Netherlands
27. Department of Clinical Epidemiology, Leiden University Medical Center, Leiden, the Netherlands

**Supplementary Figures**

**Figure 1. Calibration plots for sensitivity analyses**

**A. Subgroup of participants with inhibitor development below 50 months of age, at ITI start**


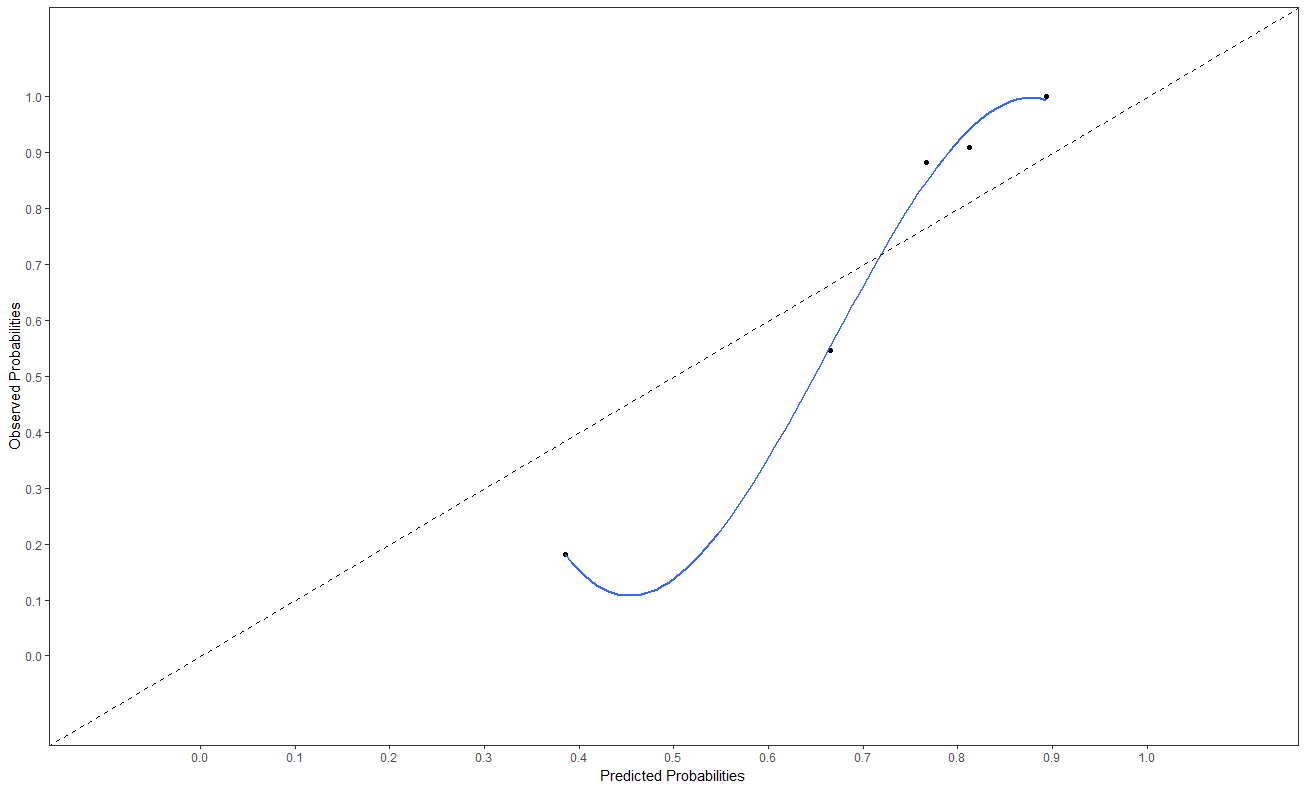


Uncorrected C-statistic 0.808 (95% CI, 0.67-0.89)

R^2^ 0.301

Corrected C-statistic 0.786

R^2^ 0.227

**B. Subgroup of participants with inhibitor development below 50 months of age, after 6 months of ITI**


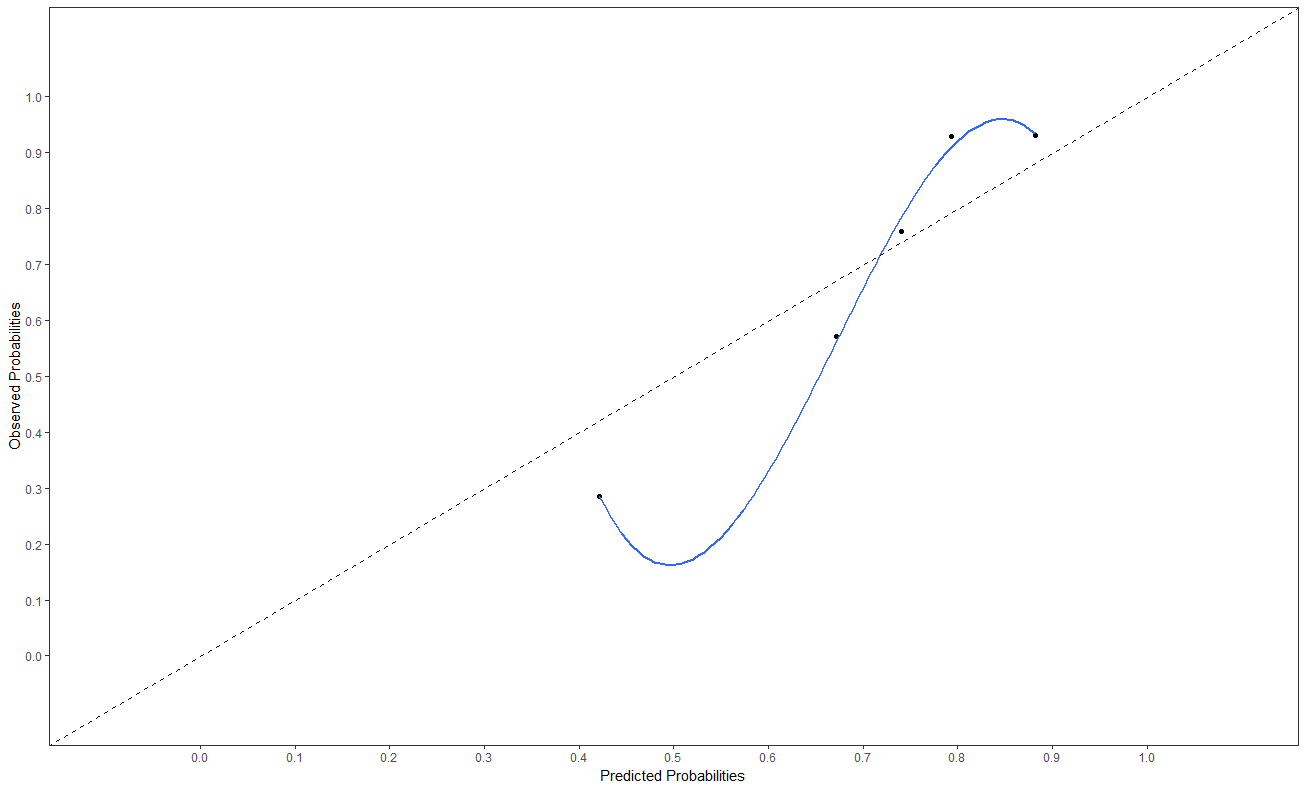


Uncorrected C-statistic 0.773 (95% CI, 0.66-0.86)

R^2^ 0.237

Corrected C-statistic 0.752

R^2^ 0.166

**C. Subgroup of participants with available data on complete success, at ITI start**


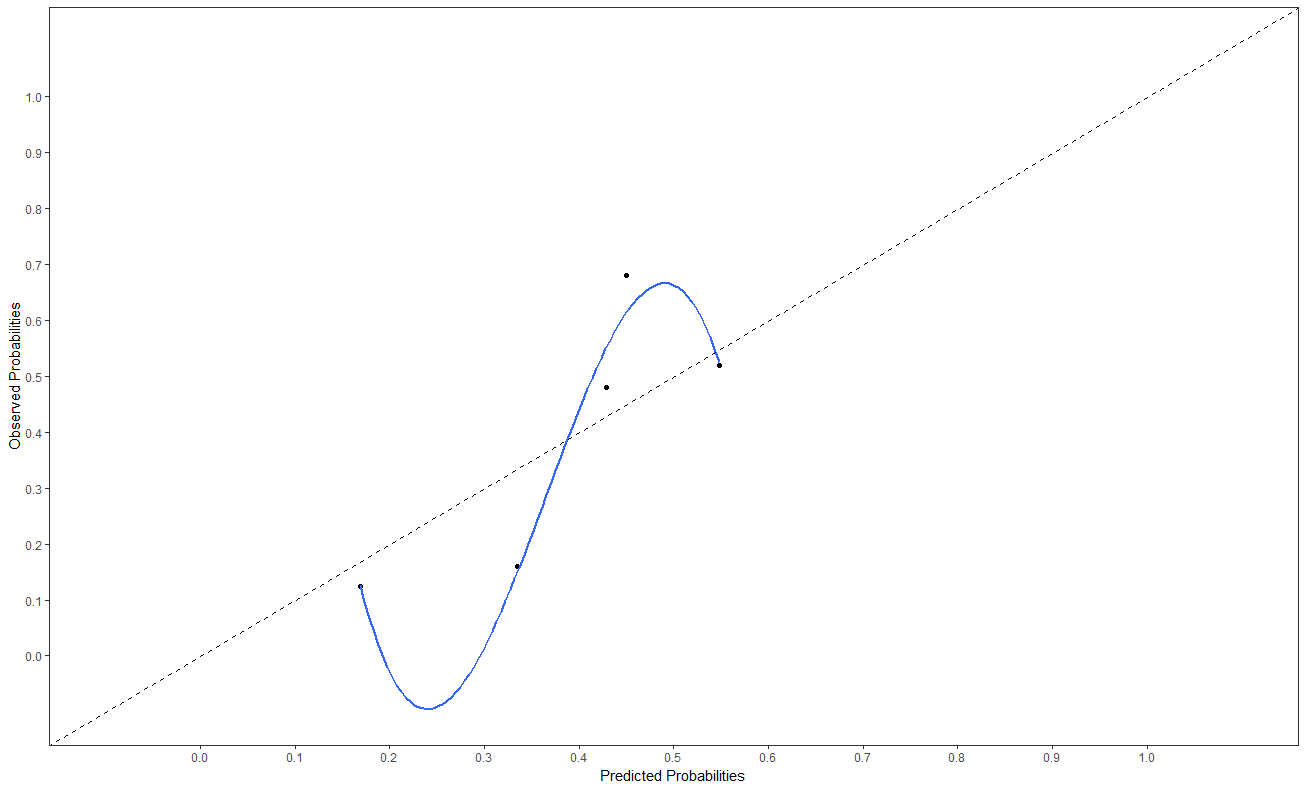


Calibration plots. These plots can be interpreted as predicted (x-axis) and observed (y-axis) probability of ITI success. Uncorrected C-statistic represents the calibration of the prediction model when applied to the data set. Corrected C-statistic shows the calibration adjusted for over-fitting using bootstrap resampling (250 runs). If the predicted probability was equal to the observed probability, the line would have followed the diagonal line.

Uncorrected C-statistic 0.657 (95% CI, 0.53-0.76)

R^2^ 0.150

Corrected C-statistic 0.647

R^2^ 0.101


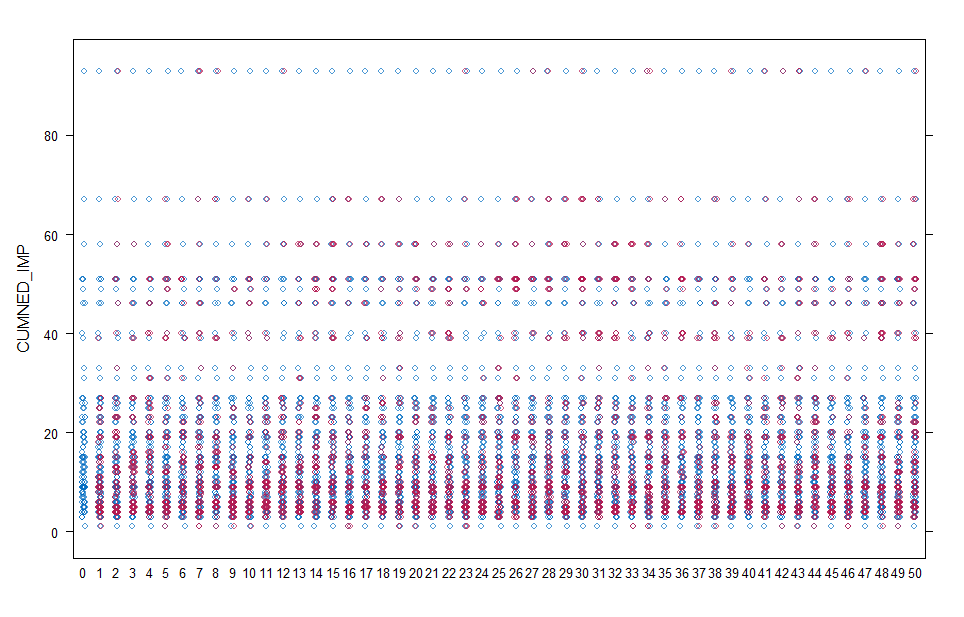

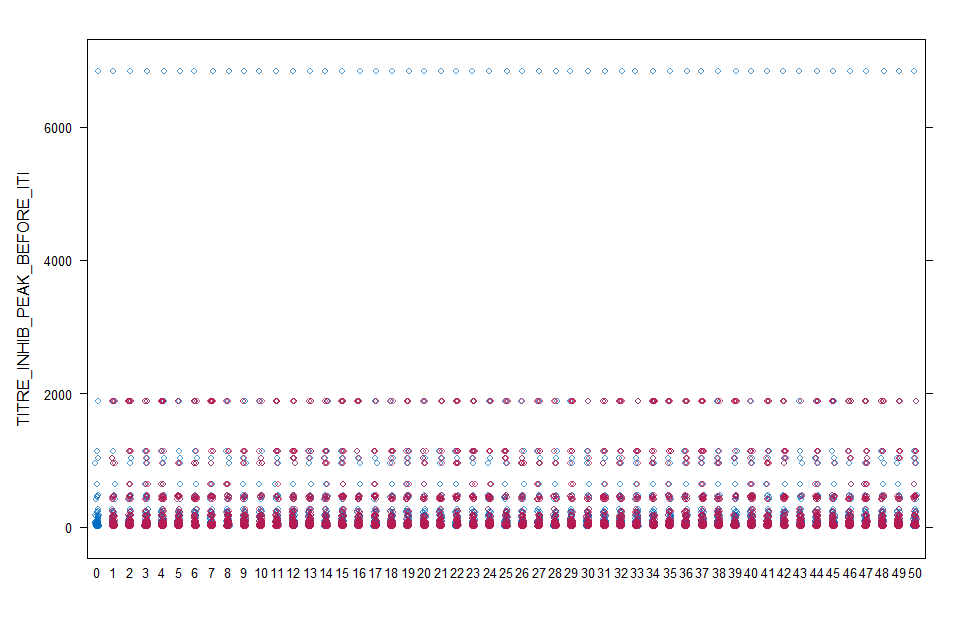
**
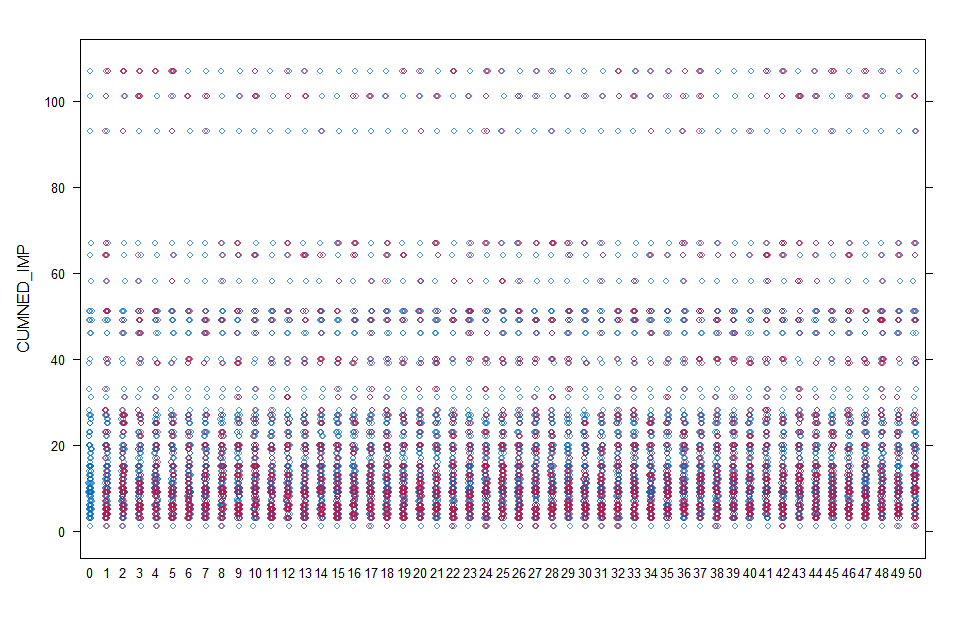
**

Trace plots were used to visually assess convergence, which was not found for variables with >10% missing data.


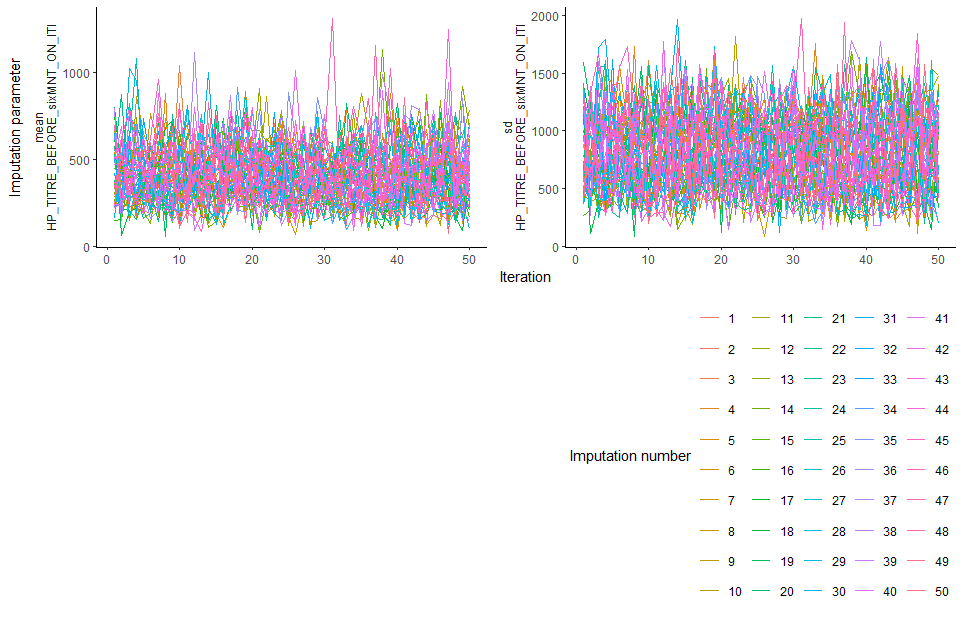


Peak inhibitor titer ever measured until 6 months on ITI


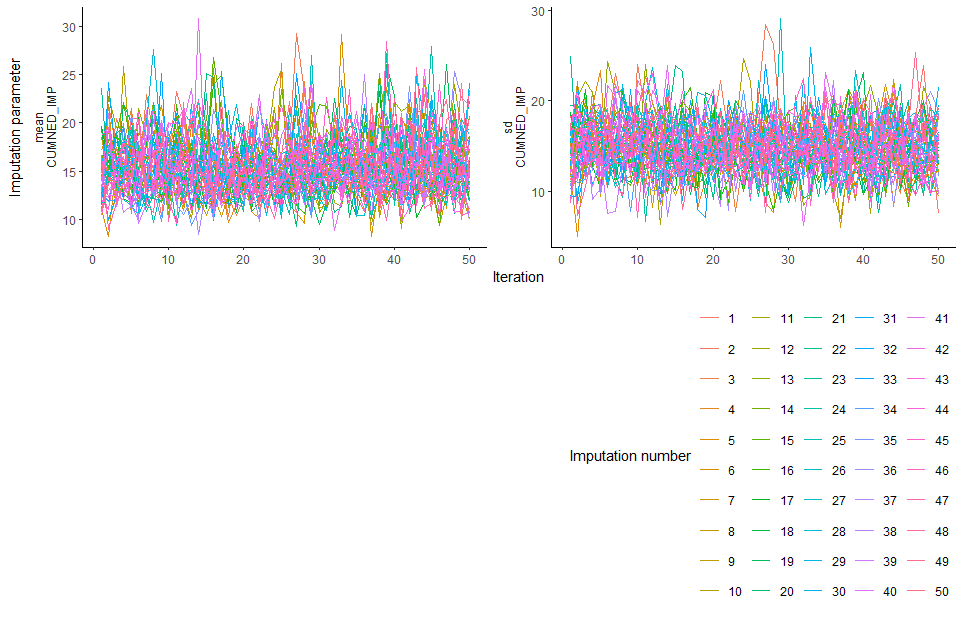


**Model B**

Cumulative number of FVIII exposure days before inhibitor development


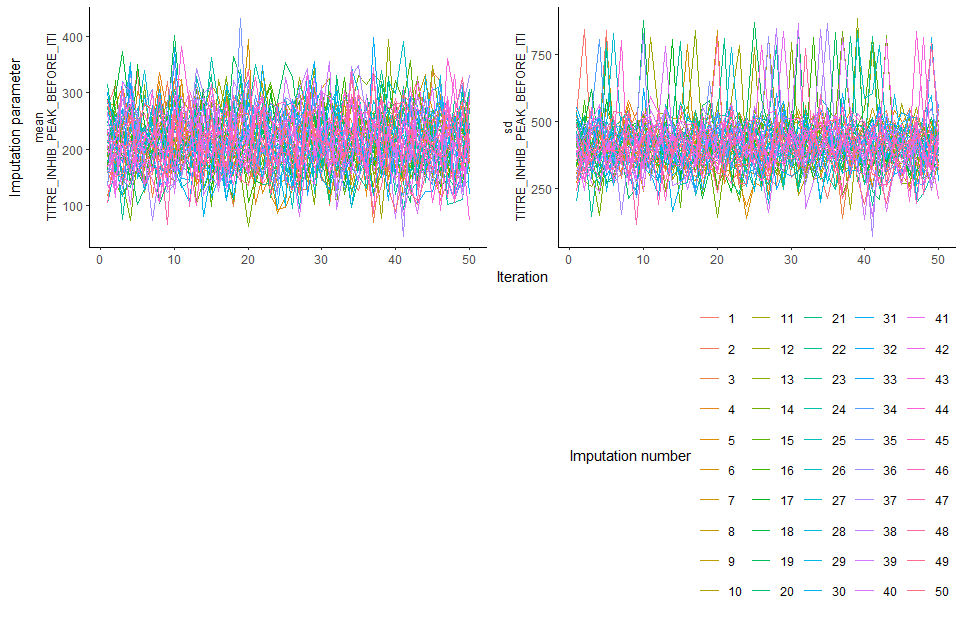


Peak inhibitor titer before ITI start


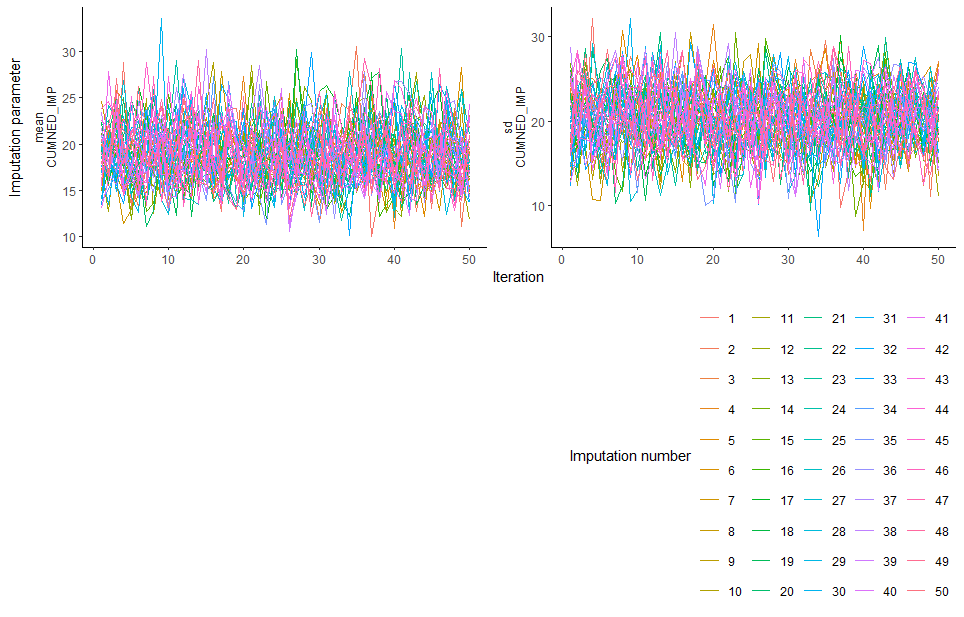


**Model A**

Cumulative number of FVIII exposure days before inhibitor development

**Figure 2. Trace plots**

**Model B**

Cumulative number of FVIII exposure days before inhibitor development

Peak inhibitor titer before ITI start

**Model A**

Cumulative number of FVIII exposure days before inhibitor development

**Figure 3. Strip plots**


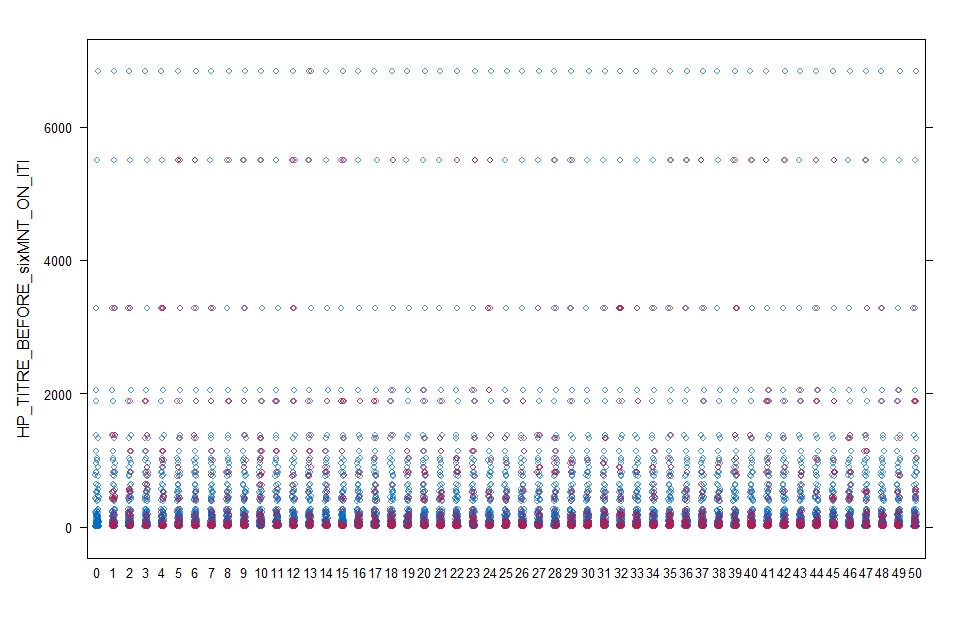
**Supplementary Tables**

Strip plots were created to compare the distribution of both imputed (red) and non-imputed (blue) individual values. The strip plots present comparable results between imputed and non-imputed data for variables with >10% missing data.

Peak inhibitor titer ever measured until 6 months on ITI

| **Table 1.** **List of participating Hemophilia Treatment Centers** |
| --- |
| **Brazil** |
| Fundação de Hematologia e Hemoterapia de Pernambuco (HEMOPE), Recife |
| Centro de Hematologia e Hemoterapia do Ceará (HEMOCE), Fortaleza |
| Centro de Hematologia e Hemoterapia de Santa Catarina (HEMOSC), Florianópolis |
| Coagulopathy Clinic, Hemocentro do Paraná (HEMEPAR), Curitiba |
| **Canada** |
| Hospital for Sick Children, Toronto |
| McMaster University, Hamilton |
| CHU Sainte-Justine, Montréal |
| Foothills Medical Center, Calgary |
| **The Netherlands** |
| Radboud university medical center, Nijmegen |
| Leiden University Medical Center, Leiden |
| Erasmus University Medical Center, Rotterdam |
| Amsterdam University Medical Centers, Amsterdam |
| Van Creveldkliniek, University Medical Center Utrecht, Utrecht |
| **Germany** |
| University Hospital Bonn, Bonn |
| **Italy** |
| IRCCS Humanitas Research Hospital, Rozzano, Milan |
| Careggi University Hospital, Florence |
| Federico II University, Naples |

| **Table 2. Univariate analysis** |  |  |
| --- | --- | --- |
| **Variable** | **OR (95% CI)** | ***p*-value** |
| **Probability of ITI success at ITI start** | | |
| Age at inhibitor development in months | 1.00 (1.00-1.01) | 0.180 |
| Inhibitor titer at detection (BU/mL) | 1.00 (1.00-1.00) | 0.091 |
| CED before inhibitor development | 1.05 (0.99-1.00) | 0.099 |
| Pre-ITI titer (BU/mL) | 1.00 (1.00-1.00) | 0.460 |
| Peak inhibitor titer before ITI start (BU/mL) | 1.00 (1.00-1.00) | 0.031 |
| Age at ITI start in months | 1.00 (1.00-1.00) | 0.505 |
| Interval between inhibitor development and ITI start in weeks | 1.00 (1.00-1.00) | 0.326 |
| ITI dose at ITI start in IU/kg/day | 1.00 (1.00-1.00) | 0.362 |
| *F8* mutation type |  |  |
| Other mutation type | *ref* | *ref* |
| Large deletion | 0.18 (0.05-0.61) | 0.006 |
| Ethnicity |  |  |
| Other ethnicity | *ref* | *ref* |
| Hispanic/Latino | 0.42 (0.17-1.03) | 0.059 |
| Family history of inhibitors |  |  |
| No family history of hemophilia | *ref* | *ref* |
| Hemophilia A, inhibitors | 0.81 (0.32-2.01) | 0.642 |
| Hemophilia A, no inhibitors | 1.28 (0.61-2.68) | 0.510 |
| Unknown | 1.99 (0.53-7.47) | 0.305 |
| FVIII product at ITI start |  |  |
| rFVIII | *ref* | *ref* |
| pdFVIII | 1.52 (0.79-2.91) | 0.210 |
| **Probability of ITI success at 6 months on ITI** | | |
| Age at inhibitor development in months | 1.00 (1.00-1.01) | 0.186 |
| Inhibitor titer at detection (BU/mL) | 1.00 (0.99-1.00) | 0.107 |
| CED before inhibitor development | 1.05 (0.99-1.11) | 0.103 |
| Pre-ITI titer (BU/mL) | 1.00 (1.00-1.00) | 0.632 |
| Peak inhibitor titer ever measured until 6 months on ITI (BU/mL) | 1.00 (1.00-1.00) | 0.054 |
| Age at ITI start in months | 1.00 (1.00-1.00) | 0.511 |
| Interval between inhibitor development and ITI start in weeks | 1.00 (1.00-1.00) | 0.318 |
| ITI dose at ITI start in IU/kg/day | 1.00 (1.00-1.00) | 0.772 |
| *F8* mutation type |  |  |
| Other mutation type | *ref* | *ref* |
| Large deletion | 0.19 (0.05-0.63) | 0.007 |
| Ethnicity |  |  |
| Other ethnicity | *ref* | *ref* |
| Hispanic/Latino | 0.43 (0.17-1.07) | 0.070 |
| Family history of inhibitors |  |  |
| No family history of hemophilia | *ref* | *ref* |
| Hemophilia A, inhibitors | 0.81 (0.31-2.14) | 0.665 |
| Hemophilia A, no inhibitors | 1.19 (0.55-2.60) | 0.656 |
| Unknown | 1.44 (0.36-5.67) | 0.604 |
| FVIII product at ITI start |  |  |
| rFVIII | *ref* | *ref* |
| pdFVIII | 1.46 (0.74-2.90) | 0.274 |
| *Abbreviations.* CED = cumulative number of FVIII exposure days, ITI = immune tolerance induction, FVIII = factor VIII, rFVIII = recombinant FVIII, pdFVIII = plasma-derived FVIII, ref = reference category, OR = odds ratio, CI = confidence interval, BU = Bethesda units, IU = international units. | | |

| **Table 3.** **Distribution before and after imputation of variables with >10% missing data before imputation** | | | | | | | |
| --- | --- | --- | --- | --- | --- | --- | --- |
| **Variables** | **Before imputation** | | |  | **After imputation (50 imputations, 50 iterations)** | | |
|  | **Total cohort** | **Success** | **Failure** |  | **Total cohort** | **Success** | **Failure** |
| **MODEL A – probability of ITI success at ITI start** | | | | | | | |
| Median CED before inhibitor development (IQR) | 12.0 (8.0-22.0) | 14.0 (9.0-25.0) | 9.0 (5.0-12.5) |  | 12.0 (7.0-22.0) | 13.0 (9.0-25.0) | 9.0 (5.0-13.8) |
| Median peak inhibitor titer before ITI start (BU/mL)(IQR) | 20.4 (7.9-76.6) | 13.1 (6.1-46.5) | 92.9 (48.0-470.0) |  | 25.6 (8.0-99.8) | 14.4 (7.0-64.0) | 99.8 (36.0-460.8) |
| **MODEL B – probability of ITI success at 6 months on ITI** | | | | | | | |
| Median CED before inhibitor development (IQR) | 12.0 (7.0-21.0) | 14.5 (9.0-23.5) | 6.0 (5.0-10.0) |  | 11.0 (6.0-20.0) | 14.0 (8.0-23.0) | 8.0 (5.0-13.0) |
| Median peak inhibitor titer ever measured until 6 months on ITI (BU/mL)(IQR) | 46.5 (10.2-193.5) | 23.7 (8.2-133.9) | 169.6 (74.5-736.0) |  | 49.6 (10.4-224.0) | 25.0 (8.8-137.8) | 160.0 (64.0-640.0) |
| *Abbreviations.* CED = cumulative number of FVIII exposure days, FVIII = factor VIII, ITI = immune tolerance induction, IQR = interquartile range, BU = Bethesda units. | | | | | | | |

| **Table 4. Characteristics per ITI outcome in sensitivity analysis, age at inhibitor development <50 months** | | | |
| --- | --- | --- | --- |
| **Characteristic** | **ITI outcome**  (N = 168) | | |
|  | **Success**  (N = 118)  n, % | **Failure**  (N = 50)  n, % | ***p-value*** |
| **PATIENT CHARACTERISTICS** |  |  |  |
| *F8* mutation type |  |  | 0.218 |
| Intron 22 inversion | 67 (70.5) | 28 (29.5) |  |
| Other intron inversion | 6 (100.0) | 0 (0.0) |  |
| Large deletion | 3 (30.0) | 7 (70.0) |  |
| Nonsense mutation | 13 (72.2) | 5 (27.8) |  |
| Small deletion or insertion | 7 (70.0) | 3 (30.0) |  |
| Missense mutation | 9 (81.8) | 2 (18.2) |  |
| Splice site mutation | 1 (100.0) | 0 (0.0) |  |
| *Missing, no mutation found/not determined* | *12 (70.6)* | *5 (29.4)* |  |
| Ethnicity |  |  | 0.523 |
| Caucasian | 94 (71.2) | 38 (28.8) |  |
| Hispanic/Latino | 7 (50.0) | 7 (50.0) |  |
| African/African-American/African-Caribbean | 4 (80.0) | 1 (20.0) |  |
| Arab/Middle Eastern | 5 (71.4) | 2 (28.6) |  |
| Asian | 4 (100.0) | 0 (0.0) |  |
| Other^‡^ | 3 (75.0) | 1 (25.0) |  |
| *Missing* | *1 (50.0)* | *1 (50.0)* |  |
| Family history |  |  | 0.465 |
| Hemophilia A, inhibitors | 13 (59.1) | 9 (40.9) |  |
| Hemophilia A, no inhibitors | 36 (76.6) | 11 (23.4) |  |
| No hemophilia A | 60 (69.0) | 27 (31.0) |  |
| Unknown family history of hemophilia A | 9 (75.0) | 3 (25.0) |  |
| **CHARACTERISTICS AT INHIBITOR DEVELOPMENT** |  |  |  |
| Age at inhibitor development in months, median (IQR) | 15.9 (10.6-24.6) | 16.5 (10.4-25.5) | 0.591 |
| *Missing* | *12 (66.7)* | *6 (33.3)* |  |
| Cumulative number of FVIII exposure days at inhibitor development, median (IQR) | 13.0 (9.0-23.0) | 9.0 (5.0-12.5) | 0.005 |
| *Missing* | *35 (61.4)* | *22 (38.6)* |  |
| Inhibitor titer at detection (BU/mL), median (IQR) | 4.3 (1.4-16.0) | 11.7 (1.3-42.3) | 0.186 |
| *Missing* | *9 (52.9)* | *8 (47.1)* |  |
| **PEAK INHIBITOR TITERS** |  |  |  |
| Peak inhibitor titer ever measured before ITI start (BU/mL), median (IQR) | 11.9 (5.9-41.6) | 81.4 (42.6-594.8) | 0.003 |
| *Missing* | *52 (61.9)* | *32 (38.1)* |  |
| Peak inhibitor titer ever measured until 6 months on ITI (BU/mL), median (IQR) | 16.0 (6.2-80.0) | 202.0 (73.1-806.3) | <0.001 |
| *Missing or people <6 months on ITI treatment* | *19 (42.2)* | *26 (57.8)* |  |
| **PRE-ITI CHARACTERISTICS** |  |  |  |
| Last inhibitor titer measured before ITI start (pre-ITI titer) (BU/mL), median (IQR) | 4.9 (2.2-11.5) | 14.0 (5.6-44.0) | 0.001 |
| *Missing* | *8 (53.3)* | *7 (46.7)* |  |
| Interval between inhibitor development and ITI start in weeks, median (IQR) | 8.5 (2.0-34.3) | 20.0 (4.5-45.0) | 0.088 |
| *Missing* | *8 (57.1)* | *6 (42.9)* |  |
| **CHARACTERITICS AT ITI START** |  |  |  |
| Age at ITI start in months, median (IQR) | 21.8 (13.7-33.6) | 22.4 (14.2-31.5) | 0.958 |
| *Missing* | *8 (72.7)* | *3 (27.3)* |  |
| FVIII product at ITI start |  |  | 0.446 |
| Recombinant FVIII | 75 (68.8) | 34 (31.2) |  |
| Plasma-derived FVIII | 41 (74.5) | 14 (25.5) |  |
| *Missing* | *2 (50.0)* | *2 (50.0)* |  |
| ITI dose at ITI start in IU/kg/day, median (IQR) | 100.0 (21.4-200.0) | 100.0 (21.4-200.0) | 0.820 |
| *Missing* | *4 (57.1)* | *3 (42.9)* |  |
| *Abbreviations.* N = number of total cohort, n = number, IQR = interquartile range, ITI = immune tolerance induction, BU = Bethesda units, FVIII = factor VIII, IU = international units. ^†^Fisher’s Exact test was performed due to small patient numbers. ^‡^ Native-American (n=2), Hispanic and Caucasian (n=1), Mexican, Hispanic and Caucasian (n=1). | | | |

| **Table 5. Characteristics per complete ITI outcome in sensitivity analysis** | | | | |
| --- | --- | --- | --- | --- |
| **Characteristic** | **Complete ITI outcome**  (N = 125) | | | |
|  | **Complete success**  (N = 49)  n (%) | **Partial success**  (N = 33)  n (%) | **Failure**  (N = 43)  n (%) | ***p-value*** |
| **PATIENT CHARACTERISTICS** |  |  |  |  |
| *F8* mutation type |  |  |  | 0.209 |
| Intron 22 inversion | 28 (42.4) | 19 (28.8) | 19 (28.8) |  |
| Other intron inversion | 1 (33.3) | 2 (66.7) | 0 (0.0) |  |
| Large deletion | 0 (0.0) | 1 (11.1) | 8 (88.9) |  |
| Nonsense mutation | 4 (30.8) | 3 (23.1) | 6 (46.2) |  |
| Small deletion or insertion | 4 (40.0) | 3 (30.0) | 3 (30.0) |  |
| Missense mutation | 2 (33.3) | 1 (16.7) | 3 (50.0) |  |
| Splice site mutation | 1 (100.0) | 0 (0.0) | 0 (0.0) |  |
| *Missing, no mutation found/not determined* | *9 (52.9)* | *4 (23.5)* | *4 (23.5)* |  |
| Ethnicity |  |  |  | 0.341 |
| Caucasian | 36 (44.4) | 19 (23.5) | 28 (34.6) |  |
| Hispanic/Latino | 5 (23.8) | 6 (28.6) | 10 (47.6) |  |
| African/African-American/African-Caribbean | 1 (25.0) | 3 (75.0) | 0 (0.0) |  |
| Arab/Middle Eastern | 3 (50.0) | 1 (16.7) | 2 (33.3) |  |
| Asian | 2 (50.0) | 2 (50.0) | 0 (0.0) |  |
| Other^‡^ | 2 (50.0) | 1 (25.0) | 1 (25.0) |  |
| *Missing* | *0 (0.0)* | *1 (33.3)* | *2 (66.7)* |  |
| Family history |  |  |  | 0.587 |
| Hemophilia A, inhibitors | 6 (40.0) | 4 (26.7) | 5 (33.3) |  |
| Hemophilia A, no inhibitors | 11 (39.3) | 8 (28.6) | 9 (32.1) |  |
| No hemophilia A | 30 (41.1) | 17 (23.3) | 26 (35.6) |  |
| Unknown family history of hemophilia A | 2 (22.2) | 4 (44.4) | 3 (33.3) |  |
| **CHARACTERISTICS AT INHIBITOR DEVELOPMENT** |  |  |  |  |
| Age at inhibitor development in months, median (IQR) | 20.0 (13.3-45.6) | 27.7 (16.3-42.0) | 23.8 (13.3-39.2) | 0.139 |
| *Missing* | *5 (41.7)* | *2 (16.7)* | *5 (41.7)* |  |
| Cumulative number of FVIII exposure days at inhibitor development, median (IQR) | 16.5 (10.0-23.0) | 17.0 (9.5-42.5) | 8.0 (5.0-11.0) | 0.013 |
| *Missing* | *15 (29.4)* | *12 (23.5)* | *24 (47.1)* |  |
| Inhibitor titer at detection (BU/mL), median (IQR) | 5.2 (1.1-16.4) | 4.3 (1.4-16.0) | 17.6 (4.6-55.3) | 0.047 |
| *Missing* | *1 (14.3)* | *1 (14.3)* | *5 (71.4)* |  |
| **PEAK INHIBITOR TITER** |  |  |  |  |
| Peak inhibitor titer ever measured before ITI start (BU/mL), median (IQR) | 22.4 (9.6-64.0) | 17.4 (7.9-77.0) | 92.9 (48.0-470.0) | <0.001 |
| *Missing* | *16 (34.8)* | *11 (23.9)* | *19 (41.3)* |  |
| **PRE-ITI CHARACTERISTICS** |  |  |  |  |
| Last inhibitor titer measured before ITI start (pre-ITI titer) (BU/mL), median (IQR) | 5.2 (2.0-11.7) | 4.0 (1.6-9.2) | 11.0 (4.8-35.5) | <0.001 |
| *Missing* | *0 (0.0)* | *2 (25.0)* | *6 (75.0)* |  |
| Interval between inhibitor development and ITI start in weeks, median (IQR) | 17.5 (2.3-90.8) | 28.5 (2.0-126.8) | 41.0 (15.0-246.3) | 0.428 |
| *Missing* | *1 (14.3)* | *1 (14.3)* | *5 (71.4)* |  |
| **CHARACTERITICS AT ITI START** |  |  |  |  |
| Age at ITI start in months, median (IQR) | 26.1 (17.1-75.4) | 38.9 (24.1-74.4) | 30.0 (21.1-88.4) | 0.426 |
| *Missing* | *4 (57.1)* | *1 (14.3)* | *2 (28.6)* |  |
| FVIII product at ITI start |  |  |  | 0.793 |
| Recombinant FVIII | 37 41.1) | 22 (24.4) | 31 (34.4) |  |
| Plasma-derived FVIII | 12 (36.4) | 10 (30.3) | 11 (33.3) |  |
| *Missing* | *0 (0.0)* | *1 (50.0)* | *1 (50.0)* |  |
| ITI dose at ITI start in IU/kg/day, median (IQR) | 25.0 (21.4-100.0) | 21.4 (21.4-50.5) | 21.4 (21.4-192.5) | 0.162 |
| *Missing* | *0 (0.0)* | *0 (0.0)* | *1 (100.0)* |  |
| *Abbreviations.* N = number of total cohort, n = number, IQR = interquartile range, ITI = immune tolerance induction, BU = Bethesda units, IU = international units. ^‡^ Native-American (n=2), Hispanic and Caucasian (n=1), Mexican, Hispanic and Caucasian (n=1). | | | | |

| **Table 6. Sensitivity analysis, final logistic regression model** | | | | | | |
| --- | --- | --- | --- | --- | --- | --- |
| **Variable** | **Original coefficients** | **Bootstrap adjusted coefficients^†^** | **Standard error** | **OR** | **95% CI** | ***p*-value** |
| **A. Probability of ITI success at ITI start including participants with age at inhibitor development <50 months** | | | | | | |
| F8 mutation type |  |  |  |  |  |  |
| Other mutation type | *ref* | *ref* | *ref* | *ref* | *ref* | *ref* |
| Large deletion | -1.470 | -1.135 | 0.813 | 0.230 | 0.05-1.15 | 0.071 |
| Ethnicity |  |  |  |  |  |  |
| Other ethnicity | *ref* | *ref* | *ref* | *ref* | *ref* | *ref* |
| Hispanic/Latino | -1.000 | -0.773 | 0.647 | 0.368 | 0.10-1.32 | 0.123 |
| CED before inhibitor development | 0.039 | 0.030 | 0.024 | 1.040 | 0.99-1.09 | 0.101 |
| Peak inhibitor titer before ITI start (BU/mL) | -0.003 | -0.002 | 0.001 | 0.997 | 0.99-1.00 | 0.051 |
| **B. Probability of ITI success at 6 months on ITI including participants with age at inhibitor development <50 months** | | | | | | |
| F8 mutation type |  |  |  |  |  |  |
| Other mutation type | *ref* | *ref* | *ref* | *ref* | *ref* | *ref* |
| Large deletion | -1.339 | -1.042 | 0.789 | 0.262 | 0.05-1.25 | 0.090 |
| CED before inhibitor development | 0.050 | 0.039 | 0.030 | 1.051 | 0.99-1.12 | 0.104 |
| Peak inhibitor titer ever measured until 6 months on ITI (BU/mL) | -0.001 | -0.001 | 0.001 | 0.999 | 1.00-1.00 | 0.030 |
| **C. Probability of complete success at ITI start including participants within complete ITI outcome definition** | | | | | | |
| Age at inhibitor development | 0.004 | 0.003 | 0.002 | 1.004 | 1.00-1.01 | 0.074 |
| Ethnicity |  |  |  |  |  |  |
| Other ethnicity | *ref* | *ref* | *ref* | *ref* | *ref* | *ref* |
| Hispanic/Latino | -1.267 | -1.009 | 0.615 | 0.282 | 0.08-0.95 | 0.040 |
| Peak inhibitor titer before ITI start (BU/mL) | -0.002 | -0.001 | 0.001 | 0.998 | 1.00-1.00 | 0.151 |
| *Abbreviations.* CED = cumulative number of FVIII exposure days, FVIII = factor VIII, ITI = immune tolerance induction, ref = reference category, OR = odds ratio, CI = confidence interval, BU = Bethesda units. †The shrinkage factor was used to obtain the bootstrap adjusted coefficients (original coefficient * shrinkage factor = bootstrap adjusted regression coefficient. The shrinkage factor was 0.773 for A, 0.778 for B, and 0.780 for C. | | | | | | |
